# Inhibiting RIP1 Improves Chronic Stress-Induced Cognitive Impairments in D-Galactose-Induced Aging Mice

**DOI:** 10.3389/fnbeh.2018.00234

**Published:** 2018-10-09

**Authors:** Wenxiang Qing, Fan Li, Xueqin Wang, Chengxuan Quan, Wen Ouyang, Qin Liao

**Affiliations:** ^1^Department of Anesthesiology, The Third Xiangya Hospital, Central South University, Changsha, China; ^2^Center for Experimental Medicine, The Third Xiangya Hospital, Central South University, Changsha, China

**Keywords:** cognitive impairment, D-galactose, neuroinflammation, RIP1, stress

## Abstract

Mounting evidence shows that chronic stress can affect both the structure and function of the brain resulting in decreased synaptic plasticity and cognitive dysfunction. Although several studies have indicated that aged brains are more vulnerable to chronic stress, it remains unknown how to prevent stress-induced memory deficits in aged animals. Neuroinflammation plays an important role in the pathogenesis of chronic stress-related brain dysfunction. Receptor-interacting protein 1 (RIP1) is a key molecule that can modulate inflammation, apoptosis, and necroptosis. Here, we investigated whether inhibiting RIP1 using necrostatin-1 during chronic stress could improve chronic stress-related brain dysfunction in D-galactose-induced aging mice. The stressed mice underwent restraint stress for 14 days. Necrostatin-1 (6.25 mg/kg) or vehicle was administered intraperitoneally once every 3 days during the stress period. Locomotor activity was tested using the open field test and cognitive function was assessed using the novel object recognition and Barnes maze tests. The hippocampus was collected to assess neuroinflammation (Iba1, IL-1α, IL-1β, TNF-α, and C1q), necroptosis [RIP1, RIP3, mixed lineage kinase domain-like (MLKL), and NF-κB], neuroplasticity (doublecortin, NR1, NR2A, NR2B, GluA1, and GluA2), and the expression of glucocorticoid and mineralocorticoid receptors. Blood samples were collected to quantify the levels of corticosterone. We found that chronic stress induced obvious memory impairment and neuroinflammation, decreased neurogenesis and GluA2 expression, and increased the expression of RIP1 and NF-κB. Inhibiting RIP1 by necrostatin-1 during chronic stress rescued the memory impairment and alleviated the pathological changes induced by stress. These suggest that inhibiting RIP1 using necrostatin-1 improves chronic stress-related brain dysfunction in D-galactose-induced aging mice. The potential mechanisms include limitation of neuroinflammation and the rescue of neurogenesis and GluA2 expression.

## Introduction

Chronic stress causes serious health problems in humans (Lupien et al., 2018). It is closely associated with brain dysfunction, including impairments of learning and memory (Sindi et al., 2017), depression (Mahar et al., 2014), anxiety (Herbison et al., 2017), and antisocial behaviors (Tielbeek et al., 2018). Moreover, stress has also been linked to late-onset Alzheimer’s disease (Machado et al., 2014). Activation of the hypothalamic–pituitary–adrenocortical (HPA) axis is the critical response during stress (Cacioppo et al., 2015). Over activation of the HPA axis and prolonged exposure to glucocorticoids are thought to exert toxic effects on the central nervous system (CNS) (Marin et al., 2011). However, moderate stress is necessary for survival, motivation, and positive striving (Epel and Lithgow, 2014). Thus, targeting HPA axis has obvious side effects during the prevention and treatment of chronic stress-related brain impairments. It is more reasonable to target non-HPA axis mechanisms in order to prevent chronic stress-related impairments.

Neuroinflammation induced by chronic stress plays an important role in chronic stress-related brain impairment. For example, Goshen et al. (2008) found that chronic mild stress increased the level of the inflammatory factor interleukin (IL)-1β in the hippocampus and induced depressive-like symptoms. Blocking IL-1 signal prevented the occurrence of depressive-like symptoms in mice that underwent chronic stress (Goshen et al., 2008). Liu et al. (2015) found that chronic unpredictable stress impaired spatial memory and hippocampal long-term potentiation, and activated the microglia. Inhibiting microglial activation using minocycline alleviated chronic stress-induced impairment of memory and hippocampal long-term potentiation (Liu et al., 2015). These studies demonstrated that limiting sterile neuroinflammation during chronic stress can prevent or alleviate brain dysfunction induced by chronic stress. However, limiting sterile neuroinflammation during chronic stress is challenging due to the blood–brain barrier and the lack of a molecular target and specific drugs.

Receptor-interacting protein 1 (RIP1) is a key molecule with multiple functions. It is a crucial upstream regulator of mixed lineage kinase domain-like (MLKL)-dependent necroptosis (Degterev et al., 2005, 2008) and caspase-8-dependent apoptosis (Holler et al., 2000) in response to stimuli such as tumour necrosis factor (TNF) and ligands of Toll-like receptors. Furthermore, RIP1 has also been implicated in the regulation of inflammation (Ting et al., 1996) independent of apoptosis and necroptosis. Recently, Christofferson et al. (2012) found a novel TNFα production pathway in an RIP1 kinase-dependent manner. The inhibition of RIP1 using necrostatin-1 completely blocked the production of TNFα (Christofferson et al., 2012). Ofengeim et al. (2017) found that RIP1 activation increased in the microglia of brains during Alzheimer’s disease. Inhibiting RIP1 activation alleviated neuroinflammation and cognitive impairments during Alzheimer’s disease (Ofengeim et al., 2017). These suggest that inhibiting RIP1 can limit inflammation signaling. Therefore, in this study, we investigated if inhibiting RIP1 could alleviate the effects of chronic stress on the function of the aged brain by limiting neuroinflammation.

Recently, several studies have indicated that the consequence of stress may be dependent on the stage of brain development (Barnum et al., 2012; Naninck et al., 2015; Kingsbury et al., 2016; Lin et al., 2016; Lesse et al., 2017). The aged brain may be more vulnerable to chronic stress (Prenderville et al., 2015). Regrettably, the impact of chronic stress in aged animals has not been studied in great detail. It remains unknown how to alleviate stress-induced memory deficits in aged animals effectively. It is well known that the injection of D-galactose provides a model for aging, and this induces and accelerates senescence in rodents (Ali et al., 2015; Rehman et al., 2017; Salehpour et al., 2017; Shwe et al., 2017). In this study, we induced aged mice via the intraperitoneal injection of D-galactose for 2 months and evaluated the effects and mechanisms of inhibiting RIP1 using necrostatin-1 on brain dysfunction induced by chronic restraint stress in D-galactose-induced aging mice. We found that inhibiting RIP1 using necrostatin-1 during chronic stress limited neuroinflammation, increased neurogenesis and GluA2 expression, and finally improved memory impairment.

## Materials and Methods

### Animals

Eight-week-old C57BL/6J male mice were used. Animals were group-housed in a quiet, uncrowded facility on a 12 h light/dark cycle (lights on at 7:00 AM, off at 7:00 PM), with *ad libitum* access to lab chow and water. Experiments were performed in accordance with the National Institutes of Health guidelines on laboratory animal welfare and approved by the Animal Ethics Committee of the Third Xiangya Hospital [Changsha, China, No. LLSC (LA) 2017-004]. All the experiments were conducted to minimize the number of animals used and their suffering.

### Experimental Groups

The mice were given intraperitoneal (i.p.) injection of D-galactose (800 mg/kg/day, Sigma-Aldrich Co., MO, United States), once daily for 60 days. Subsequently, the mice were divided into the following groups:

**Group C:** group that received natural drink and food with no intervention.
**Group C+nec-1:** group that received necrostatin-1 (6.25 mg/kg, i.p.), once every 3 days.
**Group S+DMSO:** group subjected to repeated restraint stress for 14 days, and received DMSO [2.5% dimethyl sulfoxide (DMSO) in phosphate-buffered saline (PBS)] during the stress period, once every 3 days.
**Group S+nec-1:** group subjected to repeated restraint stress for 14 days, and received necrostatin-1 (6.25 mg/kg, i.p.) during the stress period, once every 3 days.

### Administration of Drugs

#### Necrostatin-1

We first dissolved the necrostatin-1 in DMSO to prepare stock solutions with concentration 25 μg/μl. The stock solution was diluted 40 times with sterile PBS before use in order to obtain 2.5% DMSO and 0.625 μg/μl necrostatin-1. Subsequently, necrostatin-1 was administered by intraperitoneal injection (6.25 mg/kg) during the stress period, once every 3 days (Yang et al., 2017).

#### DMSO

We diluted the DMSO with sterile PBS to obtain 2.5% DMSO. This was then administered by intraperitoneal injection during the stress period, once every 3 days. The volume was similar to that of necrostatin-1.

### Repeated Restraint Stress

**FIGURE 1:**
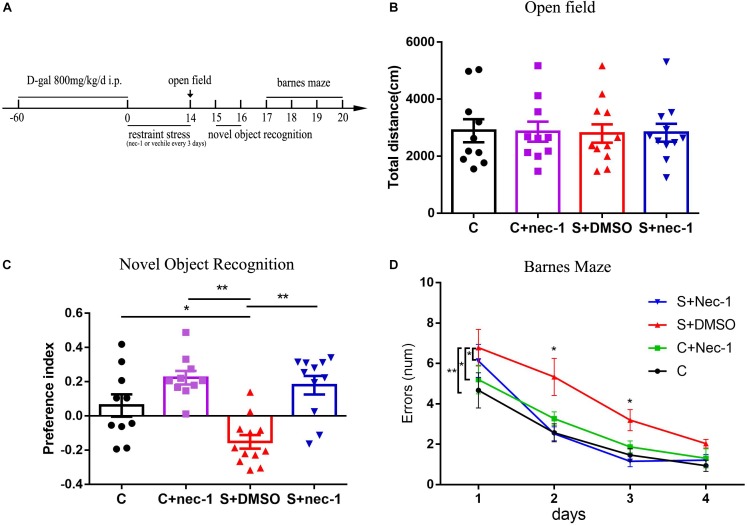
Inhibiting RIP1 using necrostatin-1 alleviated the brain dysfunction induced by chronic stress in D-galactose-induced aging mice. **(A)** Schedule of drug administration, restraint stress, and behavior test. Eight-week-old male C57BL/6J mice were intraperitoneally injected with D-galactose (800 mg/kg/day) once daily for 60 days. Subsequently, some mice were exposed to repeated restraint stress for 14 days. The mice were injected with necrostatin-1 (6.25 mg/kg) or vehicle (2.5% DMSO in PBS) once every 3 days during the stress period. **(B)** No obvious difference was detected among the four groups in the open field test [*F*_(3,39)_ = 0.01539, *p* = 0.997] (*n* = 10–12/group). Data are presented as mean ± standard error of mean (SEM). **(C)** In the novel object recognition test, the preference index of the S+DMSO group was significantly less than that of the C, C+nec-1, and S+nec-1 groups (S+DMSO vs. C: *p* < 0.05; S+DMSO vs. C+nec-1: *p* < 0.01; S+DMSO vs. S+nec-1: *p* < 0.01). **(D)** In the Barnes maze test, the errors made by the S+DMSO group were significantly more than those of the C, C+nec-1, and S+nec-1 groups (S+DMSO vs. C: *p* = 0.004; S+DMSO vs. C+nec-1: *p* = 0.0473; S+DMSO vs. S+nec-1: *p* = 0.0187, analyzed using repeated measures ANOVA). Data are presented as mean ± SEM. ^∗^*p* < 0.05; ^∗∗^*p* < 0.01; *n* = 10–12/group.

The mice were subjected to restraint stress in 50 ml conical centrifuge tubes with multiple punctures to allow ventilation. Mice were held horizontally in the tubes without food and water from 6:00 PM to 9:00 AM for 2 weeks (Liu et al., 2012). Schedules for applying restraint stress and performing cognition function tests are illustrated in **Figure 1A**.

### Open Field Test

Locomotor activity was measured in a 50 cm × 50 cm open field arena (50 cm × 50 cm × 38 cm, length × width × height). The mice were placed in the center of the apparatus and their locomotor behaviors were recorded for 5 min using a digital camera. Horizontal locomotor activity was expressed as the total ambulatory distance. The open field chamber was cleaned between trials with a 75% alcohol solution.

### Novel Object Recognition Test

Novel object recognition experiments were conducted in a 30 cm × 30 cm × 20 cm field arena. For the object recognition test, we adapted a protocol previously described in several studies (Bevins and Besheer, 2006; Leger et al., 2013; Briz et al., 2017; Volmar et al., 2017). The test comprised training and testing phases. In the training phase, two identical objects were placed equidistant from the center of the arena, and equidistant from the arena walls. The mice were placed in the arena with their heads positioned opposite to the two identical objects and allowed to freely explore the objects for 10 min. In the testing phase, mice were allowed to explore the arena that contained one of the familiar objects and a novel object for 10 min. The objects and the chamber were cleaned between trials with a 75% alcohol solution. To compare cognition in the normal adult mice (vehicle injection) with that in the D-galactose-induced aging mice (**Supplementary Material**), the interval between the training and testing phases was 24 h. In order to evaluate differences in different treatments in D-galactose-induced aging mice (**Figure 1**), a 2-h interval was used. This test is based on the spontaneous tendency of rodents to spend more time exploring a novel object than a familiar one. The exploration time of the novel object is used to assess the learning and recognition memory. The preference index was defined as (novel object investigation time–familiar object investigation time)/(novel object investigation time + familiar object investigation time).

### Barnes Maze Test

For spatial learning and memory assessment we used a 122-cm diameter Barnes maze elevated 140 cm above the floor and that contains 20 holes, each 5 cm in diameter, located evenly on the periphery of the surface. One of these holes was connected to a dark chamber called target box, which allowed the mouse to escape from an aversive bright light (200 W) (Akman et al., 2015; Wang et al., 2017). Animals were firstly placed in a black cylinder at the center of the maze for 15 s. Subsequently, the cylinder was removed and the mouse explored the maze until it found and entered the target box. It was led to the target box if it failed to do so within 3 min. Each mouse was allowed to remain in the target box for 1 min and then returned to the home cage. Each mouse underwent three trials per day with 15 min intervals between trials. The test lasted for 4 consecutive days. Between tests, the Barnes maze was cleaned with 75% alcohol solution to avoid olfactory cues. The number of incorrect hole investigation (termed error) during each trial was recorded.

### Immunohistochemistry

Mice were anesthetized with inhaled sevoflurane, and perfused transcardially with 0.01 M PBS to clear blood from body, then perfused with 4% paraformaldehyde solution to fix the tissue. Subsequently, the brains were removed and further fixed in 4% paraformaldehyde for 24 h at 4°C. Next, the brain tissues were placed sequentially in 15 and 30% sucrose in 0.01 M PBS at 4°C for dehydration. After dehydration, the brains were embedded in optimum cutting temperature compound (OCT), and coronal sections of the brain (20 μm) were serially acquired using a freezing sliding microtome (Leica CM1950, Wetzlar, Germany). For immunohistochemical detection of ionized calcium binding adaptor molecule 1 (Iba1) and DCX, free-floating sections of hippocampal tissues were washed in PBS, followed by incubation with 3% hydrogen peroxide (H_2_O_2_) in 0.1 M PBS for 10 min and 5% bovine serum albumin (BSA, Sigma, MO, United States) in 0.1 M PBS containing 0.3% Triton X-100 for 1 h. Specimens were incubated overnight at 4°C with diluted rabbit anti-Iba-1 (Wako, Japan) and rabbit anti-DCX (Cell signalling technology, Danvers, United States) both at a dilution of 1:1000. Subsequently, specimens were washed in PBS, and exposed to the corresponding biotinylated secondary antibody (Vector Laboratories, United States) at a dilution of 1:200 for 2 h. Detection of immunostaining was performed using the ABC Elite kit (Vector Laboratories, United States) and DAB kit (Beijing Zhongshan Jinqiao Biological Technology Co., Ltd., China). Finally, the specimens were dehydrated, cleared, and mounted. The photographs were obtained using a microscope (Nikon, Tokyo, Japan) and analyzed. For the Iba1 staining, the percent of activated microglia in the CA1 were determined using a previously reported method (Cerbai et al., 2012; Zhang et al., 2017b). In accordance with the report, resting microglia was defined as cells with small, round cell bodies with thin and highly ramified branches equally distributed around the cell body. Activated microglia were characterized as cells with pleomorphic bi- or tri- polar cell bodies, or spindle/rod-shaped cell bodies, with branches which were shortened, twisted, or displayed no ramification. For the DCX staining, DCX+ cells were counted in subgranular zone (SGZ) of the entire dentate gyrus (DG). The number of DCX+ cells was expressed as the number of cells in the hippocampal SGZ per section. Data were analyzed by a trained technician who was blinded to experimental conditions.

### Western Blot

Hippocampal tissues were homogenized with NP40 buffer containing 1% protease inhibitors and 1% phosphatase inhibitor (Sigma-Aldrich Co., MO, United States), followed by centrifugation at 12,000 *g* for 20 min at 4°C. The supernatant of the hippocampal homogenate was then collected. The protein concentration was determined using the bicinchoninic acid (BCA) protein assay kit (CWBio, China). Equal amounts of protein were subjected to sodium dodecyl sulfate-polyacrylamide gel electrophoresis and subsequently transferred to the PVDF membrane (Bio-Rad, CA, United States). The membranes were blocked for 1 h with 5% non-fat milk, followed by incubation with the primary antibody of anti-RIP1 (1:200, Cell signalling technology, Danvers, United States) and either RIP3, MLKL, or anti-NF-κB antibody (1:1500, Abcam, MA, United States), respectively, followed by incubation for another hour with the IRDye^®^800CW goat anti-rabbit secondary antibody (1:8000, 926-32211, LI-COR^®^, United States). Immunoblotting bands were visualized under Odyssey CLx infrared imaging systems (LI-COR^®^, United States). Protein levels were quantified by densitometry using Image J software (National Institutes of Health, MD, United States) and were normalized to GAPDH, respectively.

### Quantitative Real-Time Polymerase Chain Reaction (RT-qPCR) Assay

Total RNA was extracted using the Trizol Reagent (Invitrogen, United States) and reverse transcribed into complementary DNA using a cDNA Synthesis Kit (GeneCopoeia, United States) according to the manufacturer’s instructions. Quantitative real-time polymerase chain reaction (RT-qPCR) was performed with the mRNA qPCR mix (GeneCopoeia, United States) accordingly. Primers for all assayed genes were determined using reported sequences as listed in **Table 1**. The annealing temperature was 60°C. The reaction was performed using LightCycler^®^480II analyzer (Roche, Mannheim, Germany).

**Table 1:** Primers used for quantitative real-time PCR.

Target gene	Primers	Sequence (5′-3′)	Amplicon length (bp)
GAPDH	Forward Reverse	CATGGCCTTCCGTGTTCCTA TACTTGGCAGGTTTCTCCAGG	75
IL-1α	Forward Reverse	CCCATGATCTGGAAGAGACCA CAAACTTCTGCCTGACGAGC	99
IL-1β	Forward Reverse	TGCCACCTTTTGACAGTGATG AAGGTCCACGGGAAAGACAC	254
TNF-α	Forward Reverse	CCACCACGCTCTTCTGTCTA GAGGCCATTTGGGAACTTCTCATC	74
C1q	Forward Reverse	AGGACTGAAGGGCGTGAAAG TGGACTCTCCTGGTTGGTGA	139
NR1	Forward Reverse	CAGGTGGAGTTGAGCACCAT ATGGGACTTGAGTATGGACAGG	255
NR2A	Forward Reverse	GCGTTCAGAAGTGGTGGACT GAGGCGCTGAAGGGTTCAAG	117
NR2B	Forward Reverse	GATTCTGCATTGTGAGCCGC AGCTCGTCGACTCTCTTGGT	267
GluA1	Forward Reverse	GGACAACTCAAGCGTCCAGA CGCCACATCTGCTCTTCCATA	271
GluA2	Forward Reverse	CCCGGAAGATTGGGTACTGG ACGCTCATTCCCTTCAAGCA	176
GR	Forward Reverse	GGCAAAGGCGATACCAGGATT AGGAGCAAAGCATAGCAGGT	145
MR	Forward Reverse	GGCCAAGGTACTTCCAGGATT CCCTGGCACAGCTCATACAT	204

### Corticosterone Measurement

For corticosterone measurement, mice were anesthetized using sevoflurane after the 14-day stress. Subsequently, approximately 0.5 ml of blood was quickly collected from the ventriculus dexter. Blood samples were also collected from the control group using the same method. All the blood samples were harvested from 9:00 to 9:30 AM. The blood samples were collected into heparin-coated tubes. Subsequently, these tubes were centrifuged at 3000 *g* at 4°C for 10 min to collect the plasma. Collected plasma samples were then stored at −80°C until further analysis. A commercially available enzyme-linked immunosorbent assay (ELISA) kit obtained from Abcam (ab108821, United Kingdom) was used to quantify the levels of corticosterone in the plasma. The corticosterone measurement was performed according to the manufacturer’s instructions.

### Statistical Analysis

The data were presented as mean ± standard error of mean (SEM) and statistical analyses were performed using SPSS software version 18.0 (SPSS Inc., Chicago, IL, United States). The results of the Barnes maze test were analyzed with repeated measures analysis of variance (ANOVA). The remaining results were statistically analyzed using one-way ANOVA with *post hoc* Tukey’s multiple comparisons test. *p* < 0.05 was considered statistically significant.

## Results

### Inhibiting RIP1 Using Necrostatin-1 Alleviated Brain Dysfunction Induced by Chronic Stress in D-Galactose-Induced Aging Mice

We confirmed the artificial aging effects of D-galactose (**Supplementary Figure 1**), which is consistent with that reported in previous studies (Ali et al., 2015; Rehman et al., 2017; Salehpour et al., 2017). To assess if inhibition of RIP1 could rescue behavioral deficits of chronic stress in D-galactose-induced aging mice, we evaluated mice behaviors using the open field test, novel object recognition test, and Barnes maze test. In the open field test, there was no significant difference in total travel distance among groups C, C+nec-1, S+DMSO, and S+nec-1 [**Figure 1B**, one-way ANOVA, *F*_(3,39)_ = 0.01539, *p* = 0.9974]. In the novel object recognition test, the analysis of variance revealed an effect of group [**Figure 1C**, one-way ANOVA, *F*_(3,39)_ = 11.81, *p* < 0.001]. *Post hoc* analyses confirmed that the novel object preference of the S+DMSO group was significantly less than that of the C, C+nec-1, and S+nec-1 groups (S+DMSO vs. C: *p* < 0.05; S+DMSO vs. C+nec-1: *p* < 0.01; S+DMSO vs. S+nec-1: *p* < 0.01). There was no difference between the C+nec-1 and C groups (*p* = 0.1406). In the Barnes maze test, the repeated measures ANOVA showed significant difference in errors among groups [**Figure 1D**; *F*_(3,39)_ = 5.437, *p* = 0.0032]. *Post hoc* analyses of main effect showed that the errors made by the S+DMSO group were significantly more than those made by the C, C+nec-1, and S+nec-1 groups (S+DMSO vs. C: *p* = 0.004; S+DMSO vs. C+nec-1: *p* = 0.0473; S+DMSO vs. S+nec-1: *p* = 0.0187). There were no obvious differences between the C and C+nec-1 groups (*p* = 0.7999). Specifically, *post hoc* analyses of simple effect showed that the errors made by the S+DMSO group were significantly more than those made by the C and S+nec-1 groups on days 2 and 3 (day 2: S+DMSO vs. C: *p* = 0.013; S+DMSO vs. S+nec-1: *p* = 0.009, day 3: S+DMSO vs. C: *p* = 0.015; S+DMSO vs. S+nec-1: *p* = 0.002) (**Figure 1D**). These results suggested that necrostatin-1 alleviated the impairment of learning and memory induced by chronic stress in D-galactose-induced aging mice. Necrostatin-1 treatment had no obvious effects on the movement, learning, and memory of the artificial aging mice.

### Inhibiting RIP1 Using Necrostatin-1 Limited Neuroinflammation Induced by Chronic Stress in D-Galactose-Induced Aging Mice

**FIGURE 2:**
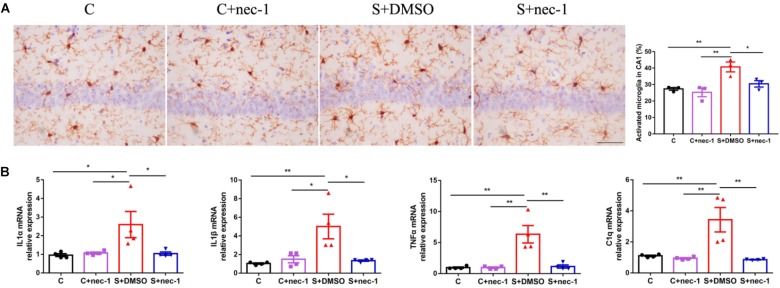
Inhibiting RIP1 using necrostatin-1 limited neuroinflammation induced by chronic stress in D-galactose-induced aging mice. **(A)** Representative images of Iba-1 staining (yellow) in the CA1. Scale bar = 50 μm. Data are expressed as the mean ± standard error of mean (SEM). ^∗^*p* < 0.05; ^∗∗^*p* < 0.01; (*n* = 3, one-way ANOVA followed by Turkey’s *post hoc* comparisons tests). **(B)** qPCR analysis of mRNA levels of inflammatory factors (IL-1α, IL-1β, C1q, and TNF-α) in the hippocampus. Data are expressed as the mean ± SEM. ^∗^*p* < 0.05; ^∗∗^*p* < 0.01; (*n* = 4, one-way ANOVA followed by Turkey’s *post hoc* comparisons tests).

Neuroinflammation contributes to cognitive dysfunction in pathological conditions (Hovens et al., 2014). Activated microglia has bigger cell bodies and shortened or twisted branches, and are usually used to identify neuroinflammation (Zhang et al., 2017b). In the present study, there were significant differences between groups based on microglia activation [**Figure 2A**, one-way ANOVA, *F*_(3,12)_ = 10.32, *p* = 0.0012]. *Post hoc* analyses showed that microglia in the cornu ammonis 1 (CA1) region of the S+DMSO mice were more activated, compared to those in the C and C+nec-1 groups (S+DMSO vs. C: *p* = 0.0039, S+DMSO vs. C+nec-1: *p* = 0.0012). Microglia activation in the S+nec-1 group significantly decreased (S+DMSO vs. S+nec-1: *p* = 0.0235) (**Figure 2A**). There was no obvious difference between the C and C+nec-1 groups (*p* = 0.8994). We also measured inflammatory factors in the hippocampus using RT-qPCR. There were significant differences between groups in the expression of IL-1α, IL-1β, TNF-α, and C1q genes [One-way ANOVA, IL-1α: *F*_(3,12)_ = 4.837, *p* = 0.0197; IL-1β: *F*_(3,12)_ = 7.342, *p* = 0.0047; TNF-α: *F*_(3,12)_ = 13.64, *p* = 0.0004; C1q: *F*_(3,12)_ = 10.74, *p* = 0.0010]. *Post hoc* analyses showed that the expression of IL-1α, IL-1β, TNF-α, and C1q genes in S+DMSO mice were significantly upregulated, compared to those in groups C (IL-1α: *p* = 0.0316, IL-1β: *p* = 0.0073, TNF-α: *p* = 0.0010, and C1q: *p* = 0.0045), C+nec-1 (IL-1α: *p* = 0.0465, IL-1β: *p* = 0.0164, TNF-α: *p* = 0.0010, and C1q: *p* = 0.0026), and S+nec-1 (IL-1α: *p* = 0.0419, IL-1β: *p* = 0.0127, TNF-α: *p* = 0.0013, and C1q: *p* = 0.0020). No significant difference was found between the C and C+nec-1 groups (IL-1α: *p* = 0.9959, IL-1β: *p* = 0.9645, TNF-α: *p* > 0.9999, and C1q: *p* = 0.9884) (**Figure 2B**). These results demonstrated that the inhibition of RIP1 limited the neuroinflammation induced by chronic stress in D-galactose-induced aging mice.

### Inhibiting RIP1 Using Necrostatin-1 Limited the Upregulation of RIP1 and NF-κB Induced by Chronic Stress in D-Galactose-Induced Aging Mice

**FIGURE 3:**
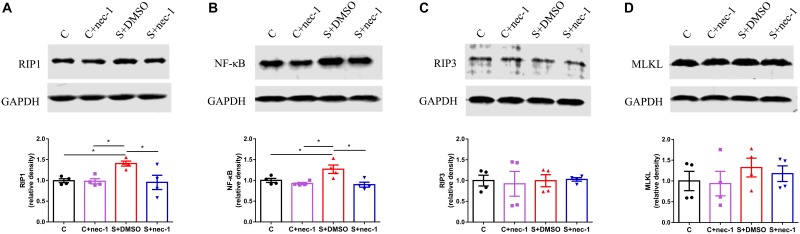
Inhibiting RIP1 using necrostatin-1 limited the upregulation of RIP1 and NF-κB induced by chronic stress in D-galactose-induced aging mice. Western blot analysis of RIP1 **(A)**, NF-κB **(B)**, RIP3 **(C)**, and MLKL **(D)** in the hippocampus. Data are expressed as the mean ± standard error of mean (SEM). ^∗^*p* < 0.05; ^∗∗^*p* < 0.01; (*n* = 4, one-way ANOVA followed by Turkey’s *post hoc* comparisons tests).

Receptor-interacting protein 1 can activate RIP3 and MLKL to promote necroptosis, and induce NF-κB-dependent inflammatory response. Thus, we detected the expression of RIP3, MLKL, and NF-κB. There were significant differences in RIP1 and NF-κB expression between groups [RIP1: **Figure 3A**, one-way ANOVA, *F*_(3,12)_ = 4.698, *p* = 0.0216; NF-κB: **Figure 3B**, one-way ANOVA, *F*_(3,12)_ = 6.789, *p* = 0.0063]. *Post hoc* analyses showed that the expressions of RIP1 and NF-κB in the S+DMSO group were significantly increased compared to those in the C (RIP1: *p* = 0.0417, NF-κB: *p* = 0.0441), C+nec-1 (RIP1: *p* = 0.0438, NF-κB: *p* = 0.0134), and S+nec-1 (RIP1: *p* = 0.0312, NF-κB: *p* = 0.0077) groups. No significant differences in the expression of RIP3 and MLKL were detected among the C, C+nec-1, S+DMSO, and S+nec-1 groups [RIP3: **Figure 3C**, one-way ANOVA, *F*_(3,12)_ = 0.06404, *p* = 0.9779; MLKL: **Figure 3D**, one-way ANOVA, *F*_(3,12)_ = 0.5465, *p* = 0.6598]. These suggested that necrostatin-1 limited the upregulation of RIP1 and NF-κB induced by chronic stress in D-galactose-induced aging mice.

### Effects of Inhibiting RIP1 Using Necrostatin-1 on Neuroplasticity

**FIGURE 4:**
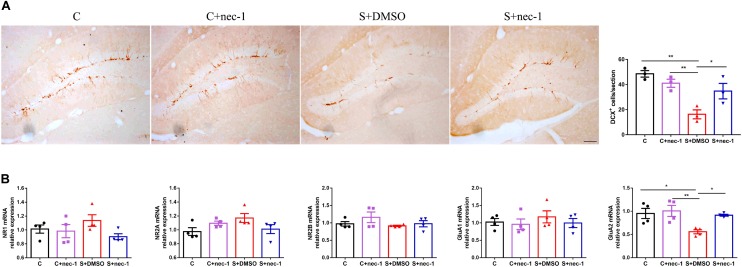
Effects of Inhibiting RIP1 using necrostatin-1 on neuroplasticity. **(A)** Representative pictures of DCX+ cells in the dentate gyrus (DG). DCX+ cells were counted in the subgranular zone (SGZ) of the entire DG. Scale bar = 50 μm. Data are expressed as the mean ± standard error of mean (SEM). ^∗^*p* < 0.05; ^∗∗^*p* < 0.01; (*n* = 3, one-way ANOVA followed by Turkey’s *post hoc* comparisons tests). **(B)** qPCR analysis of mRNA levels of NMDA receptors (NR1, NR2A, and NR2B) and AMPA receptors (GluA1 and GluA2) in the hippocampus. Data are expressed as the mean ± SEM. ^∗^*p* < 0.05; ^∗∗^*p* < 0.01; (*n* = 4, one-way ANOVA followed by Turkey’s *post hoc* comparisons tests).

Previous studies have shown that NMDA receptors, AMPA receptors, and neurogenesis are easily impaired by chronic stress (McEwen, 1999; Krugers et al., 2010; Egeland et al., 2015). Thus, we evaluated the expression of NMDA receptor subunits (NR1, NR2A, and NR2B), AMPA receptor subunits (GluA1 and GluA2), and the immature neuron marker Doublecortin (DCX). There were significant differences in DCX expression between groups [**Figure 4A**, one-way ANOVA, *F*_(3,12)_ = 9.819, *p* = 0.0015]. *Post hoc* analyses showed that DCX-positive cell numbers in the S+DMSO group significantly decreased (S+DMSO vs. C: *p* = 0.0011, S+DMSO vs. C+nec-1: *p* = 0.0086). The number of DCX-positive cells in the S+nec-1 group was significantly more than that in the S+DMSO group (S+DMSO vs. S+nec-1: *p* = 0.0495). Additionally, we noted that the numbers of AMPA receptor subunit GluA2 differed significantly between groups [one-way ANOVA, *F*_(3,12)_ = 6.437, *p* = 0.0076]. The GluA2 level in the S+DMSO group was significantly lower than that in the C (*p* = 0.0212), C+nec-1 (*p* = 0.0089), and S+nec-1 (*p* = 0.0353) groups. No obvious difference in NR1, NR2A, NR2B, and GluA1 was detected among the four groups [one-way ANOVA, NR1: *F*_(3,12)_ = 1.749, *p* = 0.2102; NR2A: *F*_(3,12)_ = 2.587, *p* = 0.1015; NR2B: *F*_(3,12)_ = 1.266, *p* = 0.3300; GluA1: *F*_(3,12)_ = 0.4093, *p* = 0.7493] (**Figure 4B**).

### Inhibiting RIP1 Using Necrostatin-1 Had No Effect on Corticosterone Level

**FIGURE 5:**
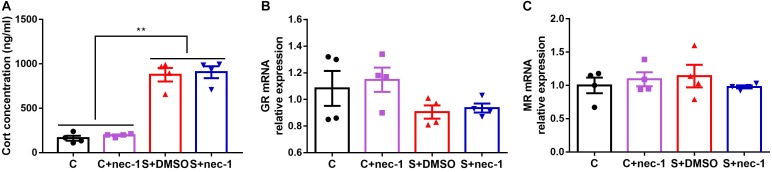
Inhibiting RIP1 using necrostatin-1 had no effect on corticosterone. **(A)** Stress elevated the corticosterone level in the blood, but necrostatin-1 had no effect on the level of corticosterone in stressed mice. **(B)** There were no significant differences among groups based on GR. **(C)** There were no significant differences among groups in terms of MR. Data are expressed as the mean ± standard error of mean (SEM). ^∗^*p* < 0.05; ^∗∗^*p* < 0.01; (*n* = 4, one-way ANOVA followed by Turkey’s *post hoc* comparisons tests).

In order to assess the effects of inhibiting RIP1 using necrostatin-1 on stress response, we measured the corticosterone level in the blood, and glucocorticoid and mineralocorticoid receptors (GR and MR) mRNA expression in the hippocampus. There were significant differences in the level of corticosterone between groups [**Figure 5A**, one-way ANOVA, *F*_(3,12)_ = 60.92, *p* < 0.0001]. *Post hoc* analyses showed that stress elevated the corticosterone level in the blood (S+DMSO vs. C: *p* < 0.0001; S+nec-1 vs. C: *p* < 0.0001; S+DMSO vs. C+nec-1: *p* < 0.0001; S+nec-1 vs. C+nec-1: *p* < 0.0001). However, necrostatin-1 had no effect on the corticosterone level in the stressed mice (S+nec-1 vs. S+DMSO: *p* = 0.9810). There were no significant differences among groups in terms of the GR and MR [GR: **Figure 5B**, one-way ANOVA, *F*_(3,12)_ = 1.913, *p* = 0.1814; MR: **Figure 5C**, one-way ANOVA, *F*_(3,12)_ = 0.4354, *p* = 0.7317].

## Discussion

In this study, we investigated the effects of chronic restraint stress on cognitive impairments in D-galactose-induced aging mice, and sought to determine whether inhibiting RIP1 using necrostatin-1 could mitigate any observed effects of stress. We found that stressed mice displayed obvious memory deficits, neuroinflammation, low neurogenesis, and GluA2 loss compared to control mice in D-galactose-induced aging mice. Administration of the RIP1 inhibitor, necrostatin-1, rescued the memory impairments and the pathological changes induced by chronic stress. These suggest that targeting RIP1 using necrostatin-1 may serve as a promising method for the prevention of impairment induced by chronic stress in aged individuals.

Chronic stress causes serious health problems in humans (Lupien et al., 2018). It is associated not only with depression (Mahar et al., 2014), anxiety (Herbison et al., 2017), and antisocial behaviors (Tielbeek et al., 2018), but also with age-related diseases such as late-onset Alzheimer’s disease (Machado et al., 2014) and late-life cognition impairment (Sindi et al., 2017). Aged individuals may show decreased resilience in response to stressors (Hidalgo et al., 2015; Prenderville et al., 2015). For instance, stress-induced reductions in neuronal dendrites in the prefrontal cortex can be reversed in young, but not in aged animals (Bloss et al., 2010). Unfortunately, there is little research on how to alleviate stress-induced memory deficits in aged animals effectively. In the present study, we established an artificial aging model. Subsequently, the aged mice were subjected to chronic restraint stress. The novel object recognition test showed that novel object preference in stressed mice significantly decreased. In the Barnes Maze test, errors made by the stressed mice were significantly increased. These results are in line with those of previous studies (Vargas-Lopez et al., 2016; Shen et al., 2018). In contrast, the memory deficits induced by chronic stress were significantly improved in mice in the necrostatin-1 treatment group (**Figure 1**). These results demonstrated that necrostatin-1 treatment efficiently prevented cognitive impairment induced by chronic stress in aging mice induced by D-galactose.

Previous studies showed that chronic stress induced obvious neuroinflammation in the brain (Bhakta et al., 2017; Kim and Won, 2017). Both the blocking of the inflammatory factor signal and the limiting of microglia activation alleviated chronic stress induced brain dysfunction (Goshen et al., 2008; Liu et al., 2015). These show that limiting sterile neuroinflammation during chronic stress could prevent or alleviate brain dysfunction induced by chronic stress. Recent studies have shown that RIP1 was a key regulator of inflammation, apoptosis, and MLKL-dependent necroptosis (Linkermann and Green, 2014; Weinlich et al., 2017). Additionally, necrostatin-1 is a highly specific and CNS-permeable inhibitor of RIP1 kinase (Ofengeim et al., 2017; Zhang et al., 2017a). Therefore, we assessed the effects of inhibiting RIP1 using necrostatin-1 on brain dysfunction induced by chronic stress in aged mice. Consistent with previous studies, we found that chronic stress induced obvious neuroinflammation (**Figure 2**) while inhibiting RIP1 using necrostatin-1 during chronic stress improved memory impairment and limited neuroinflammation (**Figures 1**, **2**). We also found that necrostatin-1 did not change the expression of RIP3 and MLKL but rather decreased the expression of RIP1 and NF-κB (**Figure 3**). These results suggested that necrostatin-1 limited the chronic stress-induced neuroinflammation possibly in an NF-κB-dependent manner.

NMDA receptor subunits (NR1, NR2A, and NR2B) and AMPA receptor subunits (GluA1 and GluA2) are closely involved in brain function, and are also important targets of chronic stress (Costa-Nunes et al., 2014; Pacheco et al., 2017; Shang et al., 2017; Arcego et al., 2018). The effects of chronic stress on NMDA and AMPA receptors vary based on the type of stress. For example, Pacheco et al. (2017) found that restraint stress (2.5 h/day) for 14 days triggered an increase in NR1 protein level in the dorsal hippocampus. Arcego et al. (2018) found that social isolation stress increased the expression of subunit NR2A of the NMDA receptor. In contrast, Costa-Nunes et al. (2014) found that social defeat and predation stress increased NR2A mRNA expression, but not the expression of NR2B and NR1 mRNA in the hippocampus of mice. In chronic unpredictable mild stress, the levels of NR2A and NR2B were significantly reduced (Shang et al., 2017), the levels of GluA2 and GluA3 were significantly elevated while the level of GluA1 was not changed (Lin et al., 2018). In the present study, we also evaluated the expression of NMDA receptor subunits (NR1, NR2A, and NR2B) and AMPA receptor subunits (GluA1 and GluA2). The expression of GluA2, but not NR1, NR2A, NR2B, and GluA1, was significantly decreased in stressed mice. In addition, we evaluated the effects of chronic stress on hippocampal neurogenesis, another important target of chronic stress (Kang et al., 2016). Chronic stress caused a significant decrease in hippocampal neurogenesis. Interestingly, chronic stress-induced loss of GluA2 expression and neurogenesis were significantly restored by necrostatin-1 treatment (**Figure 4**). Previous studies have shown that increasing hippocampal neurogenesis can improve pattern separation in object recognition (Sahay et al., 2011). Penn et al. (2017) found that GluA2 plays an important role in the maintenance of long-term potentiation and retrieval in spatial learning (Rossner et al., 2017). These results suggested that the rescue of neurogenesis and GluA2 expression were the potential mechanisms underlying the protective effect of necrostatin-1 in stressed artificial aging mice.

In addition, we measured the level of corticosterone in the blood and the expression of GR and MR mRNA in the hippocampus in order to assess the effects of inhibiting RIP1 using necrostatin-1 on stress response. Previous studies indicated that stressful events resulted in the secretion of glucocorticoids from the adrenal glands into the blood stream. Glucocorticoids regulate stress response by binding to GR and MR. Stress is known to reduce GR levels in adults through the epigenetic programming of the GR promoter. It is currently unknown whether chronic stress directly affects GR levels in aged mice (Gjerstad et al., 2018; Mifsud and Reul, 2018). In the present study, stress elevated the corticosterone level in the blood, which is consistent with the results of a previously described study (Lowrance et al., 2016). Necrostatin-1 had no effect on the corticosterone level in stressed mice. There were no significant differences among groups in terms of GR and MR (**Figure 5**). These results indicated that necrostatin-1 did not affect the stress response.

In the present study, we developed an artificial aging model using D-galactose, which is widely used in the study of accelerated aging (Ali et al., 2015; Rehman et al., 2017; Salehpour et al., 2017; Shwe et al., 2017). D-galactose induced aging has little side effect and occurs over a much shorter period of time compared to natural aging. D-galactose can accelerate the aging process, and can be used to induce senescence (Shwe et al., 2017). Although numerous studies have shown that the D-galactose-induced aging model can be used as a reliable animal model for studying mimetic aging, it is impossible to know the exact equivalent age of these mice compared to naturally aging mice. This is a limitation of the study. It is better to use naturally aging mice in further studies. Additionally, this was a correlative study. We observed various changes in the CNS in response to different treatments, but we did not confirm causally relationship of these changes. This needs to be addressed in future studies.

In summary, inhibiting RIP1 using necrostatin-1 can alleviate the cognitive impairments induced by chronic restraint stress in D-galactose-induced aging mice. The potential underlying mechanisms include limitation of neuroinflammation and the rescue of neurogenesis and GluA2 expression. Targeting RIP1 might be a novel therapeutic strategy for the treatment of chronic stress-induced cognitive impairments in aged individuals.

## Author Contributions

WO and QL designed this experiment and directed the research group in all aspects, including planning, execution, and analysis of the study. WQ was the main investigator in this study; performed the animal experiments, immunohistochemistry, western blot, qPCR, and statistical analysis; and also wrote this article, with assistance from FL, XW, and CQ. All authors have seen and approved the current version of the manuscript.

## Conflict of Interest Statement

The authors declare that the research was conducted in the absence of any commercial or financial relationships that could be construed as a potential conflict of interest.
