# Supplementary material for: Inhibiting RIP1 Improves Chronic Stress-Induced Cognitive Impairments in D-Galactose-Induced Aging Mice
Source: Front Behav Neurosci. 2018 Oct 9;12:234. doi: 10.3389/fnbeh.2018.00234 (PMC6190884; doi:10.3389/fnbeh.2018.00234)
Supplement: Supplementary file 1 [file Image_1.pdf]

Supplementary Material

Inhibiting RIP1 improves chronic stress induced cognitive impairments in D-galactose induced aging mice

Wenxiang Qing[1], Fan Li[1], Xueqin Wang[2], Chengxuan Quan[1], Wen Ouyang[1,*] and Qin Liao[1,*]

*** Correspondence:** Wen Ouyang: ouywtg@163.com and Qin Liao: zhanghaoliaoqin@163.com

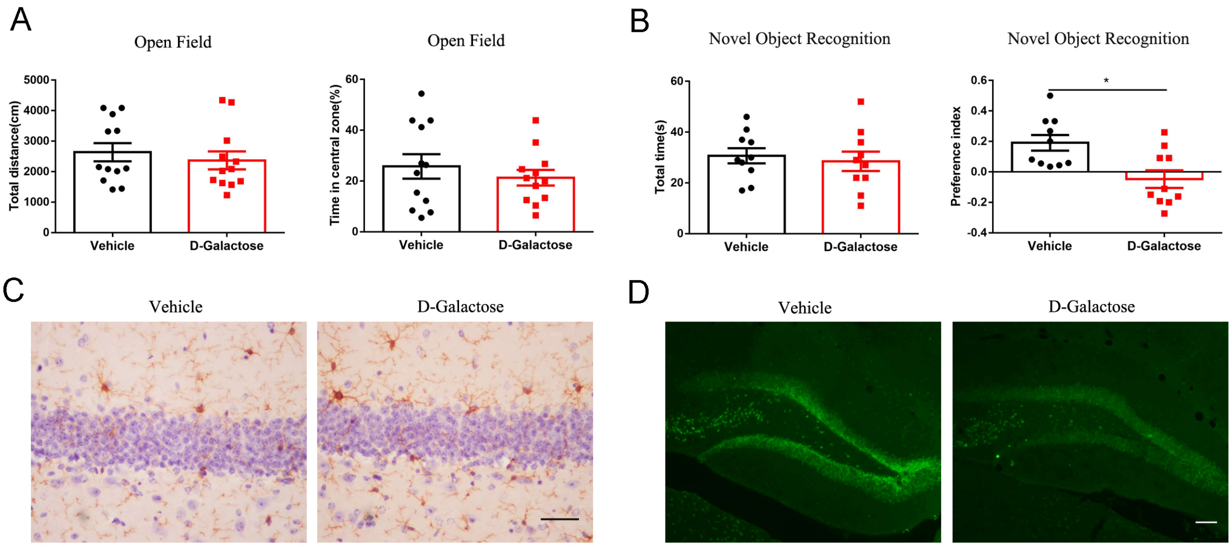

Supplementary Figure 1. Comparison of vehicle injection and D-galactose injection mice. To confirm the effect of D-Gal on accelerating senescence, we compared the D-galactose injection mice with the vehicle (PBS) injection mice. (A) No obvious difference was detected between four groups in the open field test. (B) In the novel object recognition test, Preference index of galactose group was significantly less than the NS group ($p = 0.0065$). (C) Microglia activation (Iba1) in CA1 region of the D-galactose mice was obviously increased. Scale bar = 50μm. (D) The pCREB staining indicated an obvious decline of pCREB in the D-galactose mice. Scale bar = 50μm.
